# A metadata schema for data objects in clinical research

**DOI:** 10.1186/s13063-016-1686-5

**Published:** 2016-11-24

**Authors:** Steve Canham, Christian Ohmann

**Affiliations:** 1 European Clinical Research Infrastructure Network (ECRIN), Kings Avenue, Redhill, RH16QH UK; 2 European Clinical Research Infrastructure Network (ECRIN), Kaiserswerther Strasse 70, 40477 Düsseldorf, Germany

**Keywords:** Metadata, Standards, Data sharing, Transparency, Data repositories, Identifiers, European Clinical Research Infrastructure Network, ECRIN

## Abstract

**Background:**

A large number of stakeholders have accepted the need for greater transparency in clinical research and, in the context of various initiatives and systems, have developed a diverse and expanding number of repositories for storing the data and documents created by clinical studies (collectively known as *data objects*). To make the best use of such resources, we assert that it is also necessary for stakeholders to agree and deploy a simple, consistent metadata scheme.

**Methods:**

The relevant data objects and their likely storage are described, and the requirements for metadata to support data sharing in clinical research are identified. Issues concerning persistent identifiers, for both studies and data objects, are explored.

**Results:**

A scheme is proposed that is based on the DataCite standard, with extensions to cover the needs of clinical researchers, specifically to provide (a) study identification data, including links to clinical trial registries; (b) data object characteristics and identifiers; and (c) data covering location, ownership and access to the data object. The components of the metadata scheme are described.

**Conclusions:**

The metadata schema is proposed as a natural extension of a widely agreed standard to fill a gap not tackled by other standards related to clinical research (e.g., Clinical Data Interchange Standards Consortium, Biomedical Research Integrated Domain Group). The proposal could be integrated with, but is not dependent on, other moves to better structure data in clinical research.

**Electronic supplementary material:**

The online version of this article (doi:10.1186/s13063-016-1686-5) contains supplementary material, which is available to authorized users.

## Background

Recent years have seen a welcome push towards greater transparency in clinical research. The first stage saw the drive, initially from journal editors, for the prospective registration of clinical trials [1]. The second has been the increasing pressure to publish results from all trials, at least in summary form, as exemplified by the All Trials campaign [2], as endorsed by the World Health Organisation (WHO) [3] and as facilitated by ClincalTrials.gov [4] and, in the near future, by the new European clinical trial portal [5].

But, as Vickers has recently described [6], there is an increasing consensus that a third element is required for full transparency – the source data itself, the individual participant records – so that, for example, analyses can be re-run using different methods, or secondary analyses applied, or data aggregated with that of other studies to generate more powerful meta-analyses [7–10]. But ‘raw data’ can be misleading in isolation. To fully understand the source data and the results derived from it, it is also necessary to have access to other study documents, such as protocols, clinical study reports, analysis plans and case report forms.

So, true transparency requires the availability of a wide range of clinical trial ‘data objects’, to use the generic term for anything available in an electronic format. These include published documents, documents that traditionally are private and seen only by the trial team, data sets representing summaries of results, and data sets representing the full source data. The assumption is not that all of these documents should necessarily be made public, but that, subject to proper governance and the protection of individual participant privacy, they should be made available to bona fide researchers who can provide good reasons for requesting access.

There is now very broad support for these ideas. Several major journals require a statement from authors describing their plans for data sharing [11–13]. Many funders also require a commitment to data sharing [14–16]. The pharmaceutical industry has made a public commitment to clinical trial data sharing [17], and initiatives such as the Yale Open Data Access (‘YODA’) [18] and Clinical Study Data Request (CSDR) [19] schemes provide a mechanism for researchers to submit requests for anonymised data to several drug companies. A key development has been the 2016 proposal from the International Committee of Medical Journal Editors, which stated that authors should be required ‘to share with others the de-identified individual-patient data (IPD) underlying the results presented’ [20] page 1.

A host of different data repositories have been developed to help support data-sharing initiatives. Some of these are generic, such as Datadryad [21] (recommended by the *British Medical Journal* [BMJ]), Zenodo [22] and the Dataverse Project [23]. Others are focused on particular disease areas, such as National Institute on Drug Abuse Data Share [24] for drug abuse trials and the National Database for Clinical Trials Related to Mental Illness [25], both in the United States, or the global Repository of Registered Migraine Trials [26]. A generic clinical trial data repository, for both academic and commercial users, is planned by the Multi-Regional Clinical Trials Unit (MRCT) at Harvard University [27], whilst the OpenTrials initiative, though mostly focused on metadata, is also planning to host original study data and documents when necessary [28]. These systems are in addition to the company-specific repositories already in existence, an increasing number of institution-specific data repositories that can store data objects relating to clinical research (e.g., at the University of Edinburgh [29] and the University of Nottingham [30] in the United Kingdom), and the more ‘traditional’ data repositories of clinical research material – the various publisher sites which allow access to peer-reviewed journal papers.

This does not take into account the range of material relating to clinical research, documents as well as data, that could potentially be flagged as ‘available on request’ but not moved to a designated repository at all – just stored within the systems of the original research team or department. For convenience, in this paper, the term *repository* is used to include both dedicated data and document storage facilities, explicitly labelled as a *repository*, and the information technology (IT) infrastructures used for long-term storage within an institution, when the stored material includes data objects that have been listed as potentially available to others.

As the acceptance of the need for data sharing increases, this mosaic of repositories seems likely to become more complex, even allowing for periodic attempts at collaboration or aggregation. Data and documents will therefore be split between a wide variety of repositories and storage locations, many specialised and serving particular research communities, geographic areas, or institutions, even if a few larger, more generic repositories do emerge. The files themselves are likely to be in a wide variety of different formats and file types. Gathering the relevant data, papers and documents together, in the context of a particular study or review, even identifying what is available and under what arrangements, risks becoming difficult, time-consuming and expensive.

## Methods

### Types of data objects

There is a very wide variety of ‘data objects’ that can be generated by or linked with a clinical research study, especially a clinical trial. They include the following:

- *Before the trial begins*: A protocol, funding applications, applications for ethical and regulatory approval, registry data sets, patient information sheets, consent forms, statistical analysis plan(s), a data management plan, treatment allocation plans, paper and electronic case report forms, training materials for staff, and contracts with clinical sites.
- *During the trial*: Amended versions of the protocol, amended versions of consent forms and the like, interim data sets (e.g., for safety analysis), treatment allocation records, site monitoring reports, websites and newsletters with information for the public and participants.
- *As the trial ends*: Final data sets for analysis, including subsets for sub-study or secondary endpoint analysis, a clinical study report, registry results sets, press releases, published posters, presentations and journal papers.
- *After the trial is complete*: Long-term follow-up data sets and papers, reviews and meta-analyses, re-analyses, methodological reviews, editorials and comments.

Not all of these are necessarily relevant to the primary analysis and conclusions of a study, but they can all contribute to a full understanding of the research, and, even if many are not normally public, they could all potentially be requested by other researchers.

**Fig. 1:**
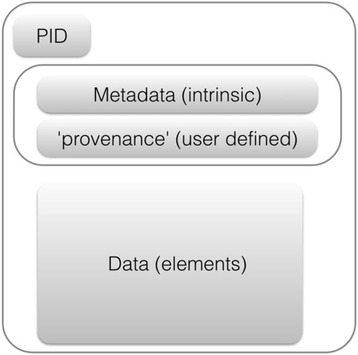
A data object’s structure (From [31].). *PID* Persistent identifier

The guiding principles for findable, accessible, interoperable and reusable data (the FAIR principles) [31] explain how, to be useful in a data-sharing context, any data object needs to have two additional properties: (a) a unique persistent identifier (PID) and (b) metadata that describes the object. The metadata is split between that which is permanent and intrinsic to the object, and that which is a function of its location and history, which could therefore differ over time (see Fig. 1).

For example, a published study protocol will probably have a digital object identifier (DOI) assigned – its PID – and the data element will be the.pdf document file itself. The associated intrinsic metadata will include the protocol title, version, authors and creation date, whereas the provenance metadata will include its uniform resource locator (URL), the organisation (publisher) hosting the document, and the accessibility regime (in this case ‘public’).

A data set made available for sharing will also need a unique identifier assigned (perhaps a DOI, perhaps a local identifier guaranteed unique by combining it with a web address), and the intrinsic metadata will need to unambiguously identify the source study, as well as the date and type of the data set. It should also reference the data object (e.g., an associated Clinical Data Interchange Standards Consortium [CDISC] operational data model [ODM] file) that contains the metadata describing the data object’s contents (which of course is distinct from the metadata describing the data object itself). The provenance metadata will again need to include the organisation currently hosting the data, and the accessibility type. If, as is likely to be the case, access is restricted, there will be no direct link to the document, but there should be some indication of how access might be gained (e.g., a URL to a website that holds details of the application process).

### A metadata proposal

A human being or a machine that wishes to identify, catalogue, search for and access a data object will need to make use of that object’s PID and the metadata. Making the best use of available clinical research data objects, given the wide variety of different repositories described above, is therefore dependent on those data objects’ having generally understood PID and metadata properties, and then reading those properties. The easiest way of simplifying that process is to have all the repositories describe their content in the same way – to develop and apply a common metadata schema for clinical research data objects.

With a common metadata scheme, systems can efficiently examine and index the data objects being stored, creating an aggregated catalogue of content across a range of related repositories (or, most ambitiously, a global catalogue across all of them). This would allow the data objects available for any study to be more easily discovered and to be described in sufficient detail for potential users to decide whether they wished to access them, or at least apply to access them.

Exactly how the metadata is organised may vary – it could be, for example, an extensible markup language (XML) file; a JavaScript object notation (JSON) file; a text document; or held within the data structures of a relational, graph or document database. Nor is there any assumption about how and where the metadata will be stored relative to the source data objects themselves. For instance, it could be in separate files stored alongside the data objects, as embedded tags in human-readable indexes of the repositories, within databases made available via an application programming interface (API), as SPARQL Protocol and RDF Query Language endpoints, or even as part of the metadata ‘injected’ into a document as can be done, for example, with .pdf files. The important point is that the data points within the schema should be consistent so that the metadata can be easily aggregated and compared, and the information within it extracted.

What should a metadata scheme for clinical research objects include? The metadata would normally need to be provided by the ‘owners’ of the data objects (i.e., those making them available for public or managed access). It should therefore be as simple and represent as light an administrative burden as possible; however, given the nature of clinical research data objects and the use cases to which such data will be put, we believe the metadata must support three main tasks:

1. Metadata should unambiguously identify the research study that the data object is about (or generated from or used within). Some data objects, such as meta-analysis documents and data sets, may refer to more than one study.
2. Metadata should characterise the research object itself – for example, its type, authorship, contents, size and language.
3. Metadata should describe where the object can be found and the access regime under which the data object is available. If not public, the regime needs to be described in sufficient detail for a potential user to be able to proceed with applying for access.

Note that the intended scope of this scheme is *all* types of protocol-driven clinical research (i.e., non-interventional studies as well as clinical trials). This is not to deny the prime importance of trials as generating the best-quality evidence, nor the fact that most of the current efforts to increase transparency are focused on clinical trials. It is simply that any metadata scheme should be flexible enough to include references to other types of research (e.g., observational, translational, using biobank data). Having a general structure in use from the beginning will be much easier than trying to adapt a more specific one later.

### Identifying the study

It would seem straightforward to describe metadata that could unambiguously identify the study or studies that a data object was ‘about’, but in fact there is no universal, consistent and PID scheme available for clinical research studies. There are various options available, but none of them are truly comprehensive. The following are some of the candidate study identifiers:

- *Registry identifiers (IDs)*: Most (but not all) clinical trials are entered prospectively into publicly accessible trial registries such as ClinicalTrials.gov. That provides them with a PID that is unique when coupled with an identifier for the registry (normally its URL or an abbreviation). But trials may have two or more such registry entries, and there is often no sense in which one is the ‘prime’ or ‘canonical’ identifier. Different people and different systems will therefore refer to a study using different registry IDs. In addition, even now, 10 years after the original decree from the International Committee of Medical Journal Editors [1] that they would not publish trial results without prior registration, a large proportion of smaller journals are still not requiring registration [32], and nearly 12% of a recent sample of published mental health trials were unregistered, along with 33% that were retrospectively registered [33]. Nevertheless, registry IDs are probably the best way currently available to identify clinical trials. Unfortunately, despite much debate about the merits of registering observational studies, only a small proportion of such studies are currently registered, and even then registration is rarely prospective [34].
- *The Universal Trial Number*: The WHO, aware of the issues of multiple registry IDs, has introduced a Universal Trial Number, or ‘UTN’ [35], but the use of this appears to be relatively limited at the moment, and it still applies only to studies that appear in registries.
- *Protocol title*: Though textual rather than an ID, all studies should have a protocol, which will have a long or ‘scientific’ title of the study on its cover. The difficulty is that the title cannot be guaranteed to be unique (unless combined with some other data – e.g., creation year, sponsor name), and it may vary slightly between different uses, such as between different versions of the protocol document.
- *Ethics identifier*: Although not all studies are registered, all studies involving people (or samples derived from living people) require ethical approval. As part of the approval process, studies almost always acquire an identifier in the ethics system. Making these identifiers accessible, and combining them with a URL for the organisation assigning them, would again provide a unique ID for the study. (If multiple ethics applications were required, then only the first need be used.) The difficulty is that this information is usually not public, so such a scheme could not be used now. It is mentioned because it is one of the few mechanisms that potentially could provide a universal index of clinical studies.

In practical terms, almost all studies will have multiple identifiers associated with them – some public, such as registry IDs and the identifiers assigned by regulatory authorities, and others internal, such as the IDs used by sponsors and funders. The best way to uniquely identify the study linked to a data object would seem to be to use the protocol title combined with whatever public identifiers are available. That data would be available to the data object ‘owners’, and it seems reasonable to expect it to be included in the associated metadata description of that object.

### Identifying the data object

Data objects published publicly (e.g., journal articles, plus some data sets and protocols in repositories) will normally already have a DOI. We propose that all publicly available clinical research data objects should have a DOI assigned, so that they have a unique identifier.

The difficulty relates to non-public data objects. We propose that, wherever possible, these data objects should also have a DOI. In particular, we would like to see DOI assignment as an integral, mandated component of the transfer process of a data object to a designated repository, even if access to the object was tightly controlled (including the scenario where the repository was institution- or company-specific). In other words, once the decision is taken to make a data object available for sharing, a DOI should be assigned, probably most easily by the original data-generating organisation. That implies an extension of current DOI use, and the process would need to be cheap and simple enough to make such assignment feasible. But it would allow any data object reference to be formulated in a consistent, unambiguous and machine-readable way, and it would provide a unique identifier for all the data objects in repositories.

But we recognise that such a development may take time and may never be applicable to all potential data objects – for instance, those still retained by the source organisation, not in a designated repository but simply in long-term storage. We therefore recommend but do not mandate a DOI for non-public data objects, because we would not like to see the need for (and the potential costs of) DOI assignment reduce the amount of material potentially available.

If a DOI is the preferred identifier but cannot be guaranteed, then the data objects will need to be identified in some other way. One approach would be to use an internal (accession) ID from a comprehensive metadata repository system, or from a confederation of such systems, that listed all the data objects known to be available. This is probably the simplest solution, though it obviously would need such metadata stores to be developed, and it introduces yet another identifier, one that would have little meaning outside the metadata repositories themselves.

A more natural approach would be to use the data object’s title, but because this will often be generic (e.g., ‘analysis data set’, ‘patient information sheet v1’), it will be necessary to combine the title with a study identifier to generate a unique identifier for the object (e.g., ‘NCT02258999\patient information sheet v1’). Unfortunately, as discussed above, there is no consistent and universally applicable method for generating a study identifier. This approach would therefore depend on first distilling and applying a universally recognised scheme for a study ID, using one of the methods already described.

We will therefore need to take a pragmatic approach in generating and assigning identifiers – for example, developing business rules that use particular identification methods in a set order, to both studies and data objects, until one of them gives a useful identifier. Both data object and study identifiers will also always need to be a composite, indicating their type and source as well as their value.

## Results

The scheme we propose is summarised by Table 1. It is based on the existing DataCite scheme for characterising data objects. (The reasons for this choice are given in the Discussion section below.) The portion of the table labelled B–E consists of intrinsic metadata that can be mapped to the DataCite scheme, whereas sections A and F represent proposed extensions to that scheme.

**Table 1:** Elements in the proposed metadata scheme for clinical research data objects

Mandatory	Recommended	Optional
A.1 Source study title^a^	A.2 Study identifier records^a^ A.3 Study topics^a^	
B.1 DOI (1) B.3 Object title	B.5 Version	B.2 Object other identifiers^a^ B.4 Object additional titles^a^
C.1 Creators^a^		C.2 Contributors^a^
D.1 Creation year		D.2 Dates^a^
E.1 Resource type general	E.2 Resource type E.3 Description^a^ E.5 Language E.6 Related identifiers^a^	E.4 Subjects (of data object)^a^
F.1 Publisher F.3 Access type F.4 Access details (2) F.5 Access contact (2) F.6 Resources^a^	F.2 Other hosting institutions^a^	F.7 Rights^a^

*DOI* Digital object identifier

(1) Mandatory for publicly accessible data objects, recommended for all others

(2) Mandatory if access is non-public

^a^May be repeating

Additional file 1 provides a more formal description of the proposed scheme and also highlights its inter-relationship with the DataCite schema. That inter-relationship is summarised in Additional file 2, whilst Additional file 3 shows how the mandatory and recommended fields in DataCite are treated within this proposal. The sections below describe the proposed scheme’s major features, using the same section headings as given in Table 1.

### A. The source study or studies

The proposal is that, for each study associated with a data object, the following data points are defined:

- A.1 Source study title (1)
  The source study title is the name of the study or studies that the data object describes, was generated by, and/or refers to (but not those it simply cites). The ‘name’ in this instance means the full or ‘scientific’ title (i.e., the title of the study protocol). For the sake of consistency, it should be the exact title as used on version 1.0 of the protocol.
- A.2 Study identifier records (0…n)
  The study identifier comprises none, one or more unique identifiers that have been assigned. There is no assumption that a study will have an identifier of a particular type. For studies entered into trial registries, these should include, at minimum, the registry ID(s), but any IDs that have been externally applied and that might be useful in identifying the study can be included. These IDs are composite. If provided, they must include not just the identifier value and type but also the assigning organisation, the scheme uniform resource identifier (URI) if there is one, and optionally the date the identifier was assigned (see Additional file 1). (Options used for identifier type and assigning organisation could be common with the lists used for DOIs, as described in B.2 below).
- A.3 Study topics (0…n)
  The study topics comprise none, one or more topic names or phrases, keywords, or classification codes describing the study or aspects of it. *Topics* is preferred to *subjects* because ‘Study subjects’ is normally understood as referring to the study participants. In the context of clinical research data objects, it makes sense to include any topic data with the study rather than the individual data objects relating to that study.

The listed topics could be free text, but it would be more useful if the text were structured (i.e., selected from a controlled vocabulary). There are a variety of such controlled vocabularies available for studies. Some refer to just a few data points, such as the International Classification of Diseases, Tenth Revision, for indicating the disease area that was the subject of the study, or Anatomical Therapeutic Chemical Classification System (ATC) categories for indicating the type of drug(s) used. More general ‘study ontologies’ are also available, such as The Cochrane Collaboration PICO ontology (patient/population/problem, intervention, comparison, outcome) [36] and, from the Biobank community, a set of study descriptors included in Minimum Information About Biobank Data Sharing ((MIABIS) [37]. There is also the Clinical Trial Registry (CTR) schema from CDISC, specifically designed to support trial registry data sets [38], which may therefore be a useful source of topic terms. To ensure that the source system is clearly identified, any use of a controlled vocabulary term should be associated with a URI that identifies the scheme (and version) being used.

Although topic data have enormous potential value in identifying related studies, in these proposals it is not mandatory. Apart from the present confusing plethora of options, there is also the problem that extracting such data retrospectively can be expensive and error-prone if not done by those most familiar with the study. One could also argue that a registry identifier, for instance, would allow the user to discover information about the study by looking up the registry data set.

In the longer term, use of more structured and consistent protocols (e.g., with CDISC CTR embedded in the Standard Protocol Items: Recommendations for Interventional Trials (‘SPIRIT’) guidelines [39]) would allow easier and more accurate extraction of structured data about studies, but we do not yet have the tools to support protocol construction in this way. If and when such tools are developed and their use becomes more common, it might be possible to mandate ‘study topic’ data of particular types, considerably enriching the metadata available.

### B–E. Data object characteristics and object identifiers

Sections B–E are heavily based on the current DataCite metadata specification [40] and so are dealt with relatively briefly.

#### B.1 Data object identifier

Data objects available publicly, such as journal articles, plus some of the data sets and protocols in repositories, should have a DOI (in line with the DataCite specification). As discussed in the Methods section above, non-public data objects should also, wherever possible, also have a DOI. If a DOI is not possible, or has not yet been assigned, then the object should be identified either by an accession number from a metadata repository system or by using the object’s name and version code, coupled with a unique identifier for the source study. The data object identifier (like study identifiers) therefore needs to be a composite, indicating its type and source as well as its value.

#### B.2 Other object identifiers

‘Other object identifiers’ refers to other unique identifiers that have been assigned to the data object in addition to its primary identifier. Again, such IDs would be composite and include the identifier type and assigning organisation, as well as its value, and optionally the identifier scheme and date of assignment. The lists used for identifier type and assigning organisation could be common with the lists used for study identifiers.

#### B.3 Object title and B.4 Additional titles

Within the context of the associated study or studies, the default name of the data object should be unique. Additional names can also be provided. If given, they are composite: the title plus one of ‘title type’ (e.g., translated title, alternative title, subtitle).

#### B.5 Version

Optionally, the version code for the data object is used. Many versions of a particular data set or document may have been created in the course of a clinical study, but the focus here is on the version or versions that are made available for sharing. The data generators will need to make that selection, though the normal expectation would be that the final version of a data object (e.g., a protocol) would be the one that was shared with others.

In some cases, multiple versions of the same document or data set could be made available, or they might be specifically requested. For instance, data sets used for the primary analysis should normally be available, as well as possible later data sets that have additional follow-up data. A protocol published before the trial began may need to be differentiated from the protocol as it existed at study end. Assuming the data objects have similar names, they will therefore need to be clearly differentiated using version codes (and relevant dates [see D.2 below] and possibly descriptions [see E.3 below]). E.6 describes how the relationship to previous or next versions can be made explicit. The form of the version coding would be as created and applied by the data generators.

#### C.1 Creators

The creators are the main personnel involved in producing the data, or the authors of a publication. It may be a set of institutional or personal names. Each name in the list is a composite element and can contain optional identifiers (e.g., Open Researcher Contributor Identification [‘ORCID’] identifiers and/or organisational affiliations, as well as the name itself).

#### C.2 Contributors

Optionally, other institutions and/or persons responsible for collecting, managing, distributing or otherwise contributing to the development of the data object can be included. If given, any contributor record is composite, with the same structure as the Creator data above, plus an additional data point specifying contributor type. The latter may need extending in the context of clinical research to include, for example, drug supplier, drug distributor, device manufacturer, central laboratory, sponsor contact, recruitment contact, principal and chief (or co-ordinating) investigator.

#### D.1 Creation year

The creation year is the year in which the object was created, expressed as four digits. Its precise definition will vary with the nature of the data object. For data sets, it will be the year of their extraction; for published documents, the year of their initial publication; and for internal documents, the year of their approval for use. Note that ‘creation year’ is intended only to provide an indicator of the time something was created (e.g., in an on-screen listing). It is not a date, which is collected and stored separately (see D.2 below).

#### D.2 Dates

None, one or more dates or date ranges that are relevant to the data object, in the standard ISO 8601 format, are used. Each date should be accompanied by a date type value that indicates what the date represents, such as accepted, available, copyrighted, collected, created, issued, submitted, updated, valid. This list (from DataCite) may need extending to better span the clinical research domain.

#### E.1 Resource type general

Resource type is one of the existing DataCite controlled list. In most cases, for clinical research data objects, the type will be ‘text’ or ‘data set’.

#### E.2 Resource type

Resource type is a description of the resource. The format is open, but the preferred format is a single term, so that a pair can be formed with the ‘resource type general’ described above (e.g., data set/census data or text/conference abstract). Existing types will need extending by a list of standard resource types for clinical research (e.g., protocol, patient information sheet, final analysis data set, quality of life data set). In practice, an expandable list would be needed (i.e., one where a user could supplement the supplied controlled vocabulary terms by free text, as and when necessary).

#### E.3 Description

The description comprises none, one or more pieces of additional general information. The format is open, but any description should be accompanied by a description type to further characterise the data: one of abstract, methods, series information, table of contents, other.

#### E.4 Subjects

Subjects comprise none, one or more subject names or phrases, keywords, classification codes describing the resource. In general, however, the recommendation is to include any subject or topic descriptors, keywords, and so forth, with the study data rather than the individual data objects (see A.3 above).

#### E.5 Language

The language is the primary language of the resource, using the International Organisation for Standardisation (ISO) language codes (e.g., EN, DE, FR).

#### E.6 Related identifiers

Related identifiers are the identifiers of related resources, which must be globally unique identifiers. Related resources will normally be data objects themselves. The record is composite and must include the identifier itself, the related identifier type and the relation type. Relation types include IsCitedBy, Cites, IsSupplementTo, IsSupplementedBy, IsContinuedBy, Continues, IsNewVersionOf, IsPreviousVersionOf, IsPartOf, HasPart, IsIdenticalTo, IsDerivedFrom and IsSourceOf.

A particularly important relationship for clinical study data is the pairing of HasMetadata-IsMetadata. Metadata in clinical research can include, for example, an ODM file or data dictionary that provides the metadata for a data set. The metadata in this context is itself a file, and as a data object in its own right, it is a ‘study data metadata data object’. This is quite distinct from the type of metadata used to describe it and all the other documents and data sets, as a data object, which is ‘data object metadata’.

#### F. Identifying location, ownership and access

The other area where the existing DataCite schema needs to be extended is in providing a full description of the access arrangements for any data object. The following data points are proposed.

#### F.1 Publisher

In this schema, this is the organisation that manages access to the document, including making the overall decision about access type (see F.3). For data, this would be the name of the organisation managing the repository. For journal papers, it is the name of the company that publishes the journal and which would normally run the primary website on which it can be accessed.

#### F.2 Other hosting institutions

Other hosting institutions are any organisations other than the publisher identified in F.1 that also host the data object within their IT infrastructure.

#### F.3 Access type

Access type is one of ‘public download’, ‘public on-screen access’, ‘restricted download’, ‘restricted on-screen access’, ‘case-by-case download’ or ‘case-by-case on-screen access’. *Restricted* means access would be dependent on membership of a predefined group, usually as determined by an authentication mechanism (e.g., username with password), such as is the case with subscription to a journal. *Case-by-case* means that there is no predefined access, but that applications for access to the data object will be considered by the object owners. *On-screen access* means that a researcher can view and process data within a specified environment but cannot download a file of the raw data, though export of the results of re-analysis would be allowed.

#### F.4 Access details (mandatory for any of the non-public access types)

*Access details* refers to a textual description of the access being offered, such as identifying the groups to which access is granted, the criteria on the basis of which a case-by-case decision would be based, or any further restrictions on on-screen access.

#### F.5 Access contact (mandatory for any of the non-public access types)

Access contact is a link to a resource that explains how access may be gained, such as how a group can be joined, and/or how application can be made for access on an individual basis. This could include an email address but more normally would be a link to a web page on the publisher’s site that would explain access procedures or provide an application pro forma.

#### F.6 Resources

Resources comprise the web-based resources that represent this data object. This is mandatory for public or restricted access objects when at least one resource should be listed. Each record would be composite and include the F.6.1 resource URL and, if downloadable, the F.6.2 resource file type (e.g., file extension or Multipurpose Internet Mail Extension [‘MIME’] type) and the F.6.3 resource size, usually in kilobytes or megabytes. The resource host would usually be obvious from the URL.

#### F.7 Rights

Rights include any intellectual property rights information for the data object, as a textual statement of the rights management associated with the resource. The URI for the specific rights management should also be given (F.7.1).

## Discussion

In constructing this proposal, we first considered the needs of potential users of any metadata scheme and then examined various existing models and metadata schemas to see if any current scheme could be used or adapted – ideally one that was already widely accepted and relatively simple to use. The difficulty is that while clinical research has generated various standards and metadata schemas, most of those are focused on the events and entities within the research process rather than on the documents and data generated by that process. For example, the CDISC has developed a range of standards allowing different types of data sets to be published in standardised formats [41], but these are focused on the data set’s contents rather than on the data sets themselves. Extensive conceptual models of protocol-based research have been developed within the Health Level 7 Reference Information Model (HL7 RIM) [42] and Biomedical Research Integrated Domain Group (BRIDG) [43] projects to facilitate interoperability between systems, but these are both highly complex and focused on the research and its regulation rather than on straightforward descriptions of its outputs.

The Organization for the Advancement of Structured Information Standards has developed standards for the component parts of electronic trial master files (eTMFs) [44], and while these do include data object properties and include a content classification scheme, it seemed to us too specifically targeted at the details of eTMF components to provide a general schema.

We also looked at more general metadata schemes for data objects, including the Dublin Core Metadata Initiative [45], the Data Documentation Initiative (DDI) [46] and DataCite [40]. The DDI schema is focused more on social sciences and the humanities than on clinical research. The Dublin Core schema is very well established, but we found that the most directly relevant scheme for our purpose was DataCite (and there are proposals to map DataCite onto the Dublin Core scheme in any case [47]). DataCite is already in widespread use, with the organisation listing over 30 members, including the British Library, the German National Library of Medicine, the Harvard University Library, the Institute of Electrical and Electronic Engineers and the Conseil Européen pour la Recherche Nucléaire [48]. Its stated purpose is to develop and support methods to locate, identify and cite data and other research objects. It therefore seemed most appropriate that the description of the data object itself be handled by the DataCite scheme, albeit with a slightly different emphasis on some components, and an extension of some part of the controlled vocabulary, to reflect the nature of data objects in clinical research.

It was then necessary to provide two logical extensions to DataCite to cover (a) the identification of the source study or studies and (b) the location, ownership and especially the nature of access to the data object, given that restricted access regimes are likely to be much more common than public access. These extensions are as described above in sections A and F in the Results section.

To keep the metadata definition as simple as possible, and focused on describing the data object, the proposed scheme requires identifiers only for the source study (or studies), such as the title and public identifiers like registry IDs. It is ‘agnostic’ about any additional study attributes or descriptors, such as methodology type, participant number, sponsor, location, chief investigator and start date. Such information could be extremely useful, and the scheme does allow for its inclusion (under A.3: Study topics above), but it does not prescribe its form. There are several reasons for this, outlined below:

1. There is no set of core attributes which by common agreement would be seen as required for all studies. The repositories being developed (e.g., the MRCT [27] and OpenTrials systems [28]) intend to list different, if overlapping, elements about the studies generating the data objects, and the same is true of different trial registries, though here at least there is a core set of 20 items specified by the WHO [49].
2. Study attributes are often not available as structured data (instead being buried within the text of protocols, and even in registry entries simply cut and pasted from there), and sometimes they are not even assigned consistently in different systems. Trying to extract, and then if necessary identify amongst different candidates, the correct attribute value can incur substantial time, effort and cost. Prospective assignment of more structured data by the researchers themselves could solve this problem, but at present the tools to support this are missing.
3. Study attributes that may be important for one type of study may not be relevant to another. Metadata for a medical device trial’s data objects could usefully indicate the type of the device(s) being tested, but this would obviously not be relevant to other types of studies. Defining a ‘core data set’ risks restricting data points to that core, when other attributes might be more significant in particular study types.
4. In most cases, at least for registered trials, registry IDs would allow navigation to a source of more detailed information about the study.

Any study attributes included would therefore be up to the metadata creators. This ensures that the proposed scheme is entirely compatible with the wide variety of systems already in existence or being developed. Having said all of that, if a particular set of study descriptor data points were to become widely available (perhaps based on the WHO core data set), it would seem wasteful not to include them in the metadata schema, as long as this did not inhibit additional attributes being added for particular types of studies.

A variety of tools could be envisaged that would allow the application of the proposed schema once an object was identified as being available for sharing, reducing the costs of creating and harvesting the metadata, better supporting the identification of relevant data objects, and thereby better promoting the overall goal of greater transparency in research. For instance, a web-based tool that prompted users for the relevant data and provided sensible choices from drop-down lists (extensible where necessary) would not only allow metadata to be created more easily but also could automatically transfer that data into storage. Linked to a generic ‘metadata repository’, it could generate both a local and a central, public metadata record, removing the need to harvest the information using API interrogation or file import.

## Conclusions

The proposed metadata scheme is an attempt to define a basic data set that is applicable to all data objects related to clinical research and addresses the need to explicitly identify the access arrangements that will apply to many of those data objects, as well as to unambiguously identify the source study. It is a relatively simple extension of a widely used existing scheme, DataCite, and is designed to be used as a foundation for implementing systems for discovering and indexing clinical research data objects. There are particular difficulties in providing unique identifiers for both the data objects and the studies with which they are linked, but these are not insurmountable. These proposals are made with the intention of initiating a debate amongst interested stakeholders, with the aim of developing a consensus on a metadata scheme that can be used throughout the clinical research community.

## Additional files

Additional file 1: Summary of the proposed metadata scheme. (DOCX 46 kb)
Additional file 2: Summary of the relationship between the proposed scheme and DataCite version 3.1. Asterisk indicates possibly repeated items. (DOCX 40 kb)
Additional file 3: Mandatory and recommended DataCite fields and how they are (a) DataCite Mandatory fields and (b) DataCite Recommended fields. Asterisk indicates field may be repeated. (DOCX 39 kb)

## Abbreviations

API: Application programming interface
ATC: Anatomical Therapeutic Chemical Classification System
BMJ: *British Medical Journal*
BRIDG: Biomedical Research Integrated Domain Group
CDISC: Clinical Data Interchange Standards Consortium
CERN: Conseil Européen pour la Recherche Nucléaire
CSDR: Clinical Study Data Request
CTR: Clinical Trial Registry
DDI: Data Documentation Initiative
DOI: Digital object identifier
eTMF: Electronic trial master file
FAIR: Findable, accessible, interoperable and reusable data
HL7 RIM: Health Level 7 Reference Information Model
ID: Identifier IPD, Individual participant (or patient) data
ISO: International Organisation for Standardisation
IT: Information technology
JSON: JavaScript object notation
MIABIS: Minimum Information About Biobank Data Sharing
MIME: Multipurpose Internet Mail Extension
MRCT: Multi-Regional Clinical Trials Unit
ODM: Operational data model
ORCID: Open Researcher Contributor Identification
PICO: Patient/population/problem, intervention, comparison, outcome
PID: Persistent identifier
SPARQL: SPARQL Protocol and RDF Query Language
SPIRIT: Standard Protocol Items: Recommendations for Interventional Trials
URI: Uniform resource identifier
URL: Uniform resource locator
UTN: Universal Trial Number
WHO: World Health Organisation
XML: Extensible markup language
YODA: Yale Open Data Access
