# Supplementary material for: A metadata schema for data objects in clinical research
Source: Trials. 2016 Nov 24;17:557. doi: 10.1186/s13063-016-1686-5 (PMC5122021; doi:10.1186/s13063-016-1686-5)
Supplement: Additional file 1: — Summary of the proposed metadata scheme. (DOCX 46 kb) [file 13063_2016_1686_MOESM1_ESM.docx]

**Additional file 1: Summary of the proposed metadata scheme**

The following sections list and briefly describe the data points in the proposed metadata scheme. Many definitions are borrowed directly from DataCite, and [DC<number>] refers to the numbering of the related item within the DC schema.

†† indicates mandatory data items, † indicates recommended data items, and other items are considered optional.

1. **Studies**

A data object will usually refer to one study, but may refer to many. *For each study associated with a data object,* it is proposed that the following data points are defined:

††**A.1 Source Study Title (1)**

The name of the study or studies that the data object describes / was generated by / refers to (but not those it simply cites). The ‘name’ in this instance means the full or ‘scientific’ title, i.e. the title of the study protocol. For consistency it should be the *exact title* as used on *version 1.0* of the protocol.

†**A.2 Study Identifiers (0...n)**

None, one or more unique identifiers that have been assigned (N.B. there is no assumption that a study will have an identifier of a particular type). For studies entered into trial registries these should include, as a minimum, the registry ID(s), but any IDs that have been externally applied, and that might be useful in identifying the study, can be included. IDs are composite: if provided, they must include an

A.2.1 identifier value (text, mandatory)

A.2.2 identifier type (selected from expandable list)

A.2.3 assigning organisation (selected from expandable list)

A.2.4 identifier scheme URL (optional)

A.2.5 date assigned (optional)

The lists used for identifier type and assigning organisation could be common with the lists used for data object identifiers, as described in B.2.

†**A.3 Study Topics (0…n)**  [DC6, DC6.1 = subject scheme, DC6.2 = scheme URI]

None, one or more topic names or phrases, keywords, or classification codes describing the study or aspects of it. Topics is preferred to ‘Subjects’ because ‘Study subjects’ is normally understood as referring to the study participants.

These are free text, but it would be much more useful if that text was linked to

A.3.1 a topic scheme, i.e. made use of a controlled vocabulary.

If a subject scheme is used the

A.3.2 topic scheme URI should also be given.

The recommendation is to include any subject topic data *with the study* rather than the individual data objects (see E.4). Provision of such data is recommended, because of its potential value in the future in linking data objects that refer to *related studies*.

**B. Object Identifiers**

**(**††**)B.1 DOI** [DC1 = identifier, DC1.1 = identifier type]

A DOI is mandated for public data objects, and recommended for all others, especially those in independent repositories, even if under controlled access.

Because a DOI cannot be guaranteed the identifier is composite with the same structure as the identifiers described in B.2. Where a DOI exists the B1.1 identifier type will be ‘DOI’. Otherwise it will be one selected from the same list as in B.2.2 below.

**B.2 Other Object Identifiers (0...n)** [DC11 = alternate identifier, DC11.1 = alt. identifier type]

None, one or more unique identifiers that have been assigned to the data object.

Such IDs are composite: if provided, they should include an

B.2.1 identifier value (text, mandatory)

B.2.2 identifier type (selected from expandable list)

B.2.3 assigning organisation (selected from expandable list)

B.2.4 identifier scheme URL (optional)

B.2.5 date assigned (optional)

This is a slight extension of the external identifier definitions in DataCite. The lists used for identifier type and assigning organisation could be common with the lists used for study identifiers, as described in A.2.

††**B.3 Object Title (1)** [DC3 = ‘Title’]

The default name of the data object. Within the context of the associated study or studies it should be unique. For a journal paper, it would simply be the title of that paper, but many other types of object will have generic titles, e.g. “final intent to treat dataset”, “patient information sheet” or even just “protocol”. Within any metadata scheme the title will therefore often need to be displayed with the study title to make it meaningful, but this is an issue for the central repository rather than the source metadata.

**B.4 Object Additional Titles (0…n)** [DC3 = title, DC3.1 = title type]

Any additional names attached to the data object. If given the item is composite: the title, plus

B.4.1 title type,

which is one of a controlled list of: Translated Title, Alternative Title, Subtitle. For translated titles the relevant language code should be given (see E.5).

†**B.5 Version (0…1)** [DC15]

The version code for the data object. Often of the form major_version.minor_version but the pattern used will be determined by the creating organisation. As stated in the DC documentation, ‘May be used in conjunction with property Description to indicate the nature and file/record range of version.’

1. **Creators and Contributors**

††**C.1 Creators (1...n)** [DC 2,2.2,2.2.1, 2.2.2 and 2.3]

The main personnel involved in producing the data, or the authors of a publication, in priority order. It may be a set of corporate / institutional or personal names.

Each name in the list is a composite element. It needs to contain a

C.1.1 name in the DataCite format (currently *family - given* for a person, or an organisation name).

C.1.2 name identifiers (optional)

Name identifiers examples include an ORCID or ISNI ID for a person, or an organisation ID from a suitable identifier system. If a name identifier is given, then for each the source

C.1.2.1 identifier scheme name,

C.1.2.2 identifier scheme URI (optional)

should also be provided.

The metadata can also include, for a person name, one or more

C1.3 organizational or institutional affiliations of that person, as free text.

**C.2 Contributors (0…n)** [DC 7,7.1, 7.2,7.3, 7.3.1, 7.3.2 and 7.4]

Optionally, the metadata can include other institutions and / or persons responsible for collecting, managing, distributing, or otherwise contributing to the development of the resource. If given, any contributor record is composite. It needs to contain name data, in exactly the same format as the Creator data described above:

C.2.1 name in the format *family, given* for a person, or an organisation name.

C.2.2 name identifiers (optional)

C.2.2.1 identifier scheme name,

C.2.2.2 identifier scheme URI (optional)

C2.3 organizational or institutional affiliations of that person, as free text.

The contributor record must also include an indication of the

C.2.4 Contributor type.

DataCite lists the following types: Contact Person, Data Collector, Data Curator, Data Manager, Distributor, Editor, Funder, Producer, Project Leader, Project Manager, Project Member, Registration Agency, Registration Authority, Related Person, Researcher, Research Group, Rights Holder, Sponsor, Supervisor, Work Package Leader, and Other, (detailed definitions of these are available within the DC documentation.

All of the above seem potentially relevant to clinical research, but additional types may be required that are specific to the domain, for example Drug supplier, Drug distributor, Device manufacturer, Central laboratory, Recruitment contact, and Chief Investigator. The option of free text will probably be needed for situations where none of the listed types apply.

1. **Dates**

††**D.1 Creation year (1)** [DC5 = publication year]

The year in which the object was created, expressed as 4 digits. The DC schema uses ‘PublicationYear’ but publication is not assumed in this scheme for all data objects (only potential availability). The ‘creation year’ refers to the time point when the data object was viewed as ‘finished’ by its creators. Its precise definition will vary with the nature of the data object. For data sets it will be the year of their extraction, for published documents the year of their initial publication, and for internal documents the year of their approval for use.

**D.2 Dates** **(0…n)** [DC8 = date, DC8.1 = dateType]

None, one or more dates or date ranges that are relevant to the work, expressed in ISO 8601 format. If given, each date should be accompanied by a

D.2.1 dateType item.

The controlled vocabulary list within the DC schema includes Accepted, Available, Copyrighted, Collected, Created, Issued, Submitted, Updated, Valid, and the DC documentation includes definitions of these. It is suggested that this list is supplemented by Published (electronically), Published (on paper) and Extracted (for data sets).

1. **General Data Object Attributes**

††**E.1 Resource Type General (1)**  [DC10.1]

One of the existing DataCite controlled list: Audiovisual, Collection, Dataset, Event, Image, Interactive Resource, Model, Physical Object, Service, software, Sound, Text, Workflow, Other. In most cases, for clinical research data objects, the type will be ‘Text’ or ‘Dataset’. It may be that other general resource types could be identified and used in the particular context of clinical research.

†**E.2 Resource Type (0…1)** [DC10]

A description of the resource. The format is open, but the preferred format is a single term, so that a pair can be formed with the ‘Resource Type General’ described above, e.g. Dataset/Census Data or Text/Conference Abstract. For published text types the standard Resource Types can be found in the CASRAI list at [http://dictionary.casrai.org/Output_Types.](http://dictionary.casrai.org/Output_Types)

These types will need extending by a list of standard document types for clinical research (e.g. protocol, patient information sheet, CSR) and also by a common set of clinical research datasets (e.g. final analysis dataset, safety dataset, quality of life dataset). In practice an expandable list would be needed – i.e. one where a user could supplement the supplied controlled vocabulary terms by free text, as and when necessary.

N.B. Data objects like datasets that themselves require detailed description would usually be related to the data objects representing their metadata (see E.6).

†**E.3 Description (0…n)** [DC17, DC17.1 = descriptionType]

Additional general information that does not fit in any of the other categories. The format is open. Any description should be accompanied by a

E.3.1 Description Type

to further characterise the data, one of Abstract, Methods, Series Information, Table of Contents, Other.

**E.4 Subjects (0…n)** [DC6, DC6.1 = subjectScheme, DC6.2 = SchemeURI]

None, one or more subject names or phrases, keywords, classification codes describing the resource. Free text in format, but often linked to a

E.4.1 subject scheme and

E.4.2 scheme URI.

For published documents keywords may already be available. In general, however, the recommendation is to include any subject / topic descriptors, keyword etc., with the study data rather than the individual data objects (see A.3).

†**E.5 Language (0…1)** [DC9]

The primary language of the resource. Values should be taken from ISO language codes, e.g. de, fr.

†**E.6 Related Identifiers (0…n)** [DC12, DC12.1, 12.2,12.3, 12.4, 12.5]

These are the Identifiers of related resources, which must be globally unique identifiers. Related resources will normally be data objects themselves. The record is composite and must include:

E.6.1 The identifier itself

E.6.2 The related Identifier Type.

The current available values include*:* ARK, arXiv, bibcode, DOI, EAN13, EISSN, Handle, ISBN, ISSN, ISTC, LISSN LSID, PMID, PURL, UPC, URL, URN.

E.6.3 The relation Type.

Current available values include*:* IsCitedBy, Cites, IsSupplementTo, IsSupplementedBy, IsContinuedBy, Continues, HasMetadata, IsMetadataFor, IsNewVersionOf, IsPreviousVersionOf, IsPartOf, HasPart, IsReferencedBy, References, IsDocumentedBy, Documents, IsCompiledBy, Compiles, IsVariantFormOf, IsOriginalFormOf, IsIdenticalTo, IsReviewedBy, Reviews, IsDerivedFrom, and IsSourceOf.

If the relation type is one of HasMetadata / Is Metadata, then either or both records should also include the name of the

E.6.4 related Metadata Scheme,

E.6.5 the scheme URI, and

E.6.6. the scheme type,

e.g. XSD, DDT, Turtle, (or in the clinical research context, CDISC ODM or Define XML).

If the HasMetadata / IsMetadata relation type is used the expectation is that all metadata data objects would be made available with the ‘target’ data object. In this case providing the related identifier information is mandatory.

1. **Identifying Location, Ownership and Access**

††**F.1 Publisher (1)**  [DC4]

In the DataCite schema the publisher is ‘The name of the entity that holds, archives, publishes prints, distributes, releases, issues, or produces the resource.’ Specifically, in this schema, it is the organisation that *manages access* to the document, including making the overall decision about access type, (see F3). For journal papers it is *not* the journal name, but the name of the company that publishes the journal, and which would normally run the primary web site on which it can be accessed.

†**F.2 Other Hosting Institutions (0…n)** [DC7 (one of the listed options)]

Any organisations other than the publisher identified in F1 that also host the data object within their IT infrastructure. This is recommended, but it is accepted that in some cases the organisation providing the source metadata may not know of every other host organisation. It may therefore be up to the metadata repository to aggregate this data point, once it has identified all hosts for a particular data object.

††**F.3 Access Type (1)**

One of ‘public download’, ‘public on-screen access’, ‘restricted download’, ‘restricted on-screen access’, ‘case by case download’ and ‘case by case on-screen access’.

*Restricted* means access would be dependent on membership of a predefined group, usually as determined by an authentication mechanism (e.g. username + password), for example as is the case with subscription to a journal. *Case by case* means that there is no predefined access but that applications for access to the data object will be considered by the object owners. *On-screen access* means that a researcher can view and process data within a specified environment but cannot download a file of the raw data, though export of the results of re-analysis would be allowed.

**(**††**) F.4 Access Details (1)**

*Mandatory for any of the non-public access types.*

A textual description of the access being offered, for example identifying the groups to which access is granted, or the criteria on which a case-by-case decision would be based.

**(**††**) F.5 Access Contact (1)**

*Mandatory for any of the non-public access types.*

A link to a resource that explains how access may be gained, e.g. how a group can be joined, and / or how application can be made for access on an individual basis.

Could use an email address but not normally that of an individual – it would be the address of a shared mailbox that was accessed by the relevant publisher staff. A URL to a web page on the publisher’s site, for example explaining the access procedures or even including an application proforma, would be preferable.

**(**††**) F.6 Resources (1…n)** [DC 13, DC14]

The web based resources that represent this data object. Mandatory for public or restricted access objects, when at least one resource should be listed.

Each record would be composite and include the

F.6.1 resource URL, and, if downloadable, the

F.6.2 resource file type (e.g. file extension or MIME type) and the

F.6.3 resource size, usually in KB or MB.

The resource host would usually be obvious from the URL.

**F.7 Rights (0…n)**  [DC16, DC16.1]

Any intellectual property rights information for the data object. A textual statement of the rights management associated with the resource. To quote the DataCite specification, ‘Use the complete title of a license and include version information if applicable. Example: Creative Commons, Attribution 3.0 Germany License’.

F.7.1 The **URI** for the specific rights management should also be given.
