# Supplementary material for: A metadata schema for data objects in clinical research
Source: Trials. 2016 Nov 24;17:557. doi: 10.1186/s13063-016-1686-5 (PMC5122021; doi:10.1186/s13063-016-1686-5)
Supplement: Additional file 2: — Summary of the relationship between the proposed scheme and DataCite version 3.1. Asterisk indicates possibly repeated items. (DOCX 40 kb) [file 13063_2016_1686_MOESM2_ESM.docx]

**Additional file 2:** Summary of the relationship between the proposed scheme and DataCite v3.1 (* indicates possibly repeated items)

| Proposed Metadata Scheme | DataCite v3.1 |
| --- | --- |
| A.1 Source Study Title* | None |
| A.2 Study Identifier records*  A.2.1 identifier value  A.2.2 identifier type  A.2.3 assigning organisation  A.2.4 identifier scheme URL (optional)  A.2.5 date assigned (optional) | None, *but analogous (but at study level) to*  *1 Identifier*   - 1. *identifier type (= DOI)*   *11 Alternate identifier*  *11. 1 Alternate identifier type* |
| A.3 Study Topics*  A.3.1 a subject scheme  A.3.2 scheme URI | 6 Subjects (analogous but at study level)  6.1 subject scheme,  6.2 scheme URI |
| B.1 Object DOI  1.1. identifier type (often = DOI) | 1 Identifier   - 1. identifier type (= DOI) |
| B.2 Object External Identifiers*  B.2.1 identifier value  B.2.2 identifier type  B.2.3 assigning organisation  B.2.4 identifier scheme URL  B.2.5 date assigned | 11 Alternate identifier  11. 1 Alternate identifier type |
| B.3 Object Title | 3 Title |
| B.4 Object Additional Titles*  B.4.1 Title type | 3 Title  3.1 Title type |
| B.5 Version | 15 Version |
| C.1 Creators*  C.1.1 name in the format *family, given* for a person, or an organisation name.  C.1.2 name identifiers (optional)  C.1.2.1 identifier scheme name,  C.1.2.2 identifier scheme URI (optional)  C1.3 organizational or institutional affiliations | 2  2.1 Creator name  2.2 Name identifier  2.2.1 Name identifier scheme  2.2.2 Scheme URI  2.3 Affiliation |
| C.2 Contributors*  C.2.1 name for a person, or an organisation  C.2.2 name identifiers (optional)  C.2.2.1 identifier scheme name,  C.2.2.2 identifier scheme URI (optional)  C2.3 organizational or institutional affiliations  C.2.4 Relationship type. | 7 Contributor  7.3 Contributor name  7.3 Name identifier  7.3.1 Name identifier scheme  7.3.2 Scheme URI  7.4 Affiliation  7.1 Contributor type |
| Proposed Metadata Scheme | **DataCite v3.1** |
| D.1 Creation year | 5 Publication year *(not exactly the same*) |
| D.2 Dates*  D.2.1 Date Type item | 8 Date  8.1 Date Type |
| E.1 Resource Type General | 10.1 Resource type general |
| E.2 Resource Type | 10 Resource type |
| E.3 Description*  E.3.1 Description Type | 17 Description  17.1 Description Type |
| E.4 Subjects*  E.4.1 Subject scheme  E.4.2 Subject URI | 6 Subject  DC6.1 Subject scheme,  DC6.2 Scheme URI |
| E.5 Language | 9 Language |
| E.6 Related Identifiers*  E.6.1 The identifier itself  E.6.2 The related Identifier Type.  E.6.3 The relation Type.  E.6.4 related Metadata Scheme,  E.6.5 the scheme URI  E.6.6. the scheme type | 12 Related identifier  12.1 Related identifier type  12.2 Relation type  12.3 Related metadata scheme  12.4 Scheme URI  12.5 Scheme type |
| F.1 Publisher | 4 Publisher |
| F.2 Other Hosting Institutions* | 7 Contributor (one of the listed options) |
| F.3 Access Type | *None* |
| F.4 Access Details | *None* |
| F.5 Access Contact | *None* |
| F.6 Resources*  F.6.1 Resource URL  F.6.2 Resource file type  F.6.3 Resource size | 14 Format  13 Size |
| F.7 Rights*  F.7.1 URI | 16 Rights  16.1 Rights URI |
