# Supplementary material for: A metadata schema for data objects in clinical research
Source: Trials. 2016 Nov 24;17:557. doi: 10.1186/s13063-016-1686-5 (PMC5122021; doi:10.1186/s13063-016-1686-5)
Supplement: Additional file 3: — Mandatory and recommended DataCite fields and how they are (a) DataCite Mandatory fields and (b) DataCite Recommended fields. Asterisk indicates field may be repeated. (DOCX 39 kb) [file 13063_2016_1686_MOESM3_ESM.docx]

**Additional file 3:** Mandatory and recommended DataCite fields and how they are

1. DataCite Mandatory fields (* indicates field may be repeated)

| Mandatory DataCite fields | Proposal fields |
| --- | --- |
| 1 Identifier (DOI) | B.1 Object DOI (mandated for public objects) |
| 2 Creator* | C.1 Creator* (mandated) |
| 3 Title | B.3 Object Title (mandated) |
| 4 Publisher | F.1 Publisher (mandated) |
| 5 Publication year | D.1 Creation year (not exactly the same*,* mandated) |

All DataCite mandatory fields are included as mandatory fields in the proposal, except the object identifier, which is mandated only for public data objects. The recommendation is that the identifier should be a DOI where possible.

1. DataCite Recommended fields (* indicates field may be repeated)

| Proposed Metadata Scheme | Proposal fields |
| --- | --- |
| 6 Subject* | Deprecated: *A.3 Study Topics** preferred instead |
| 7 Contributor* | C.2 Contributors |
| 8 Date* | D.2 Dates* |
| 10 Resource type | E.1 Resource Type General (mandated)  E.2 Resource Type (recommended) |
| 12 Related identifier* | E.6 Related Identifiers* |
| 17 Description* | E.3 Description* (recommended) |
| 18 Geolocation | Not used (URL used instead) |

Of the 7 recommended fields in DataCite all but Geolocation have an equivalent in the proposed scheme. However object Subject(s) is deprecated and Subject topics is the preferred alternative. Description and resource types are also recommended within the proposal; contributors, dates and related identifiers are optional.
